# Terminal-selective C(sp^3^)–H borylation of unbranched alkanes enabled by intermolecular radical sampling and LMCT photocatalysis

**DOI:** 10.1093/nsr/nwae105

**Published:** 2024-03-19

**Authors:** Zhexuan Lei, Jie Wu

**Affiliations:** Department of Chemistry, National University of Singapore, Singapore; Department of Chemistry, National University of Singapore, Singapore

**Figure 1.**
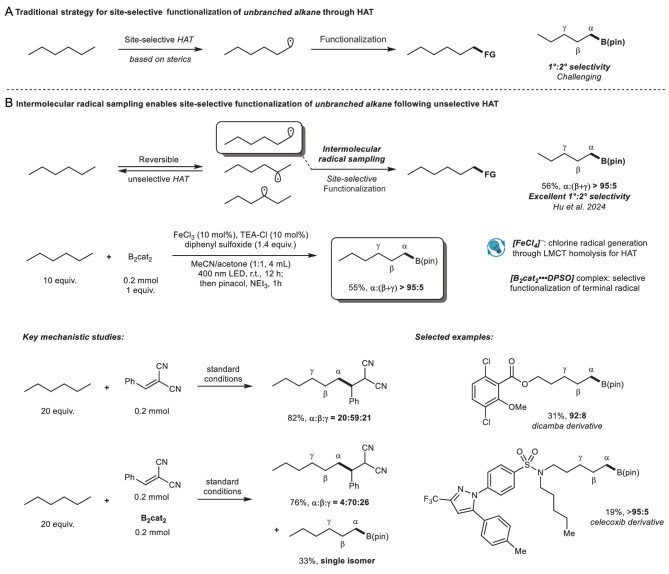
Site-selective borylation enabled by intermolecular radical sampling.

The selective functionalization of C(sp^3^)–H bonds with subtle differences in alkanes is highly sought after due to its atom- and step-efficiency. The activation of C(sp^3^)–H bonds through hydrogen atom transfer (HAT) has been extensively exploited to produce a value-added product from unfunctionalized material [1,2]. Despite the advancements in HAT chemistry, the activation of inert C(sp^3^)–H bonds under mild conditions requires a highly reactive HAT reagent, often exhibiting poor site selectivity. Prominent studies on selective C(sp^3^)–H functionalization through HAT have been reported, mainly exploiting the differences in steric hinderance of various C(sp^3^)–H bonds, achieving site-selective HAT followed by functionalization (Fig. 1A) [3–5]. However, these methods rarely produce ideal site selectivity with straight-chain substrates (seldomly exceeding 50% for terminal C(sp^3^)–H) where the steric difference between primary and secondary C(sp^3^)−H bonds is subtle.

‘Radical sampling’ involves selecting the preferred radical, among a series of radical species, to undergo the consequence step. Recently, Xu *et al*. reported a radical-mediated site-selective 1,4-translocation of cyano group (–CN), enabled by reversible and unselective HAT followed by site-selective –CN translocation to the C-4 radical due to favorable five-membered radical intermediate [6]. This provides another potential strategy for site-selective C(sp^3^)–H functionalization, which begins with an unselective HAT, followed by an ‘intermolecular radical sampling’ process, during which functionalization of the desired radical takes place selectively to form the product, while the unfunctionalized radicals revert to the substrate through the reverse HAT (Fig. 1B).

Recently reporting in *Science*, Hu *et al*. described a terminal-selective C(sp^3^)–H borylation of unbranched alkanes enabled by the ‘intermolecular radical sampling’ process (Fig. 1B) [7]. Using iron photocatalysis, chlorine radicals are generated through homolysis and exploited as a HAT reagent, generating a series of alkyl radicals through unselective HAT. Next, the primary radical is selectively borylated through a ‘radical sampling’ process to give the terminal-functionalized product. Specifically, diphenyl sulfoxide was used as a terminal oxidant, while forming a complex with B_2_cat_2_, which is responsible for the site selectivity by distinguishing different radicals based on steric hindrance. Using this method, the borylation of hexane could be achieved with excellent terminal selectivity (>95:5). This protocol can be easily applied to various substrates with straight alkyl chains, showing great functional group tolerance and high site selectivity.

The mechanism of this transformation was thoroughly studied through a series of delicate control experiments. The key evidence for the proposed pathway involves using a mixture of 2-benzylidnemalononitrile (BMN) and B_2_cat_2_ as the alkyl radical trapper (Fig. 1B). When only BMN was used, functionalization with preference for the secondary site was observed. When a mixture of BMN and B_2_cat_2_ was employed, the alkylation still preferred secondary C(sp^3^)–H bonds, while the borylation happened solely on the terminal position. These results proved that the initial chlorine radical-enabled HAT is unselective, while the borylation is terminal-selective.

This work represents a significant advancement in site-selective functionalization of C(sp^3^)–H bonds in unbranched alkanes. A novel strategy involving an intermolecular radical sampling process has been introduced to selectively capture the targeted radical. This concept can be readily extended to other types of C(sp^3^)–H functionalization by developing corresponding radical sampling reagents.
